# From phyllosphere to insect cuticles: silkworms gather antifungal bacteria from mulberry leaves to battle fungal parasite attacks

**DOI:** 10.1186/s40168-024-01764-6

**Published:** 2024-02-26

**Authors:** Pengfei Zhao, Song Hong, Yuekun Li, Haimin Chen, Hanchun Gao, Chengshu Wang

**Affiliations:** 1 Key Laboratory of Insect Developmental and Evolutionary Biology, CAS Center for Excellence in Molecular Plant Sciences, Shanghai Institute of Plant Physiology and Ecology, Chinese Academy of Sciences, Shanghai, 200032 China; 2 School of Life Science and Technology, ShanghaiTech University, Shanghai, 201210 China; 3 CAS Center for Excellence in Biotic Interactions, University of Chinese Academy of Sciences, Beijing, 100049 China

**Keywords:** Silkworm, Cuticular microbiotas, Cross-kingdom transfer, Antifungal lysozyme, Probiotic

## Abstract

**Background:**

Bacterial transfers from plants to insect herbivore guts have been well investigated. However, bacterial exchanges between plant phyllospheres and insect cuticles remain unclear, as does their related biological function.

**Results:**

Here, we report that the cuticular bacterial loads of silkworm larvae quickly increased after molting and feeding on the white mulberry (*Morus alba*) leaves. The isolation and examination of silkworm cuticular bacteria identified one bacterium *Mammaliicoccus sciuri* that could completely inhibit the spore germination of fungal entomopathogens *Metarhizium robertsii* and *Beauveria bassiana*. Interestingly, *Ma. sciuri* was evident originally from mulberry leaves, which could produce a secreted chitinolytic lysozyme (termed Msp1) to damage fungal cell walls. In consistency, the deletion of *Msp1* substantially impaired bacterial antifungal activity. Pretreating silkworm larvae with *Ma. sciuri* cells followed by fungal topical infections revealed that this bacterium could help defend silkworms against fungal infections. Unsurprisingly, the protective efficacy of Δ*Msp1* was considerably reduced when compared with that of wild-type bacterium. Administration of bacterium-treated diets had no negative effect on silkworm development; instead, bacterial supplementation could protect the artificial diet from *Aspergillus* contamination.

**Conclusions:**

The results of this study evidence that the cross-kingdom transfer of bacteria from plant phyllospheres to insect herbivore cuticles can help protect insects against fungal parasite attacks.

Video Abstract

**Supplementary Information:**

The online version contains supplementary material available at 10.1186/s40168-024-01764-6.

## Background

Many insects are herbivores that feed on or inside plants. The cross-kingdom transfers or exchanges of bacteria from plant phyllospheres to insects or vice versa have been reported [1–4]. The plant phyllosphere bacteria ingested by insects can colonize insect guts to facilitate insect detoxification, nutrient acquisition, and/or survival [3–5]. In addition to the gut microbiotas, the ectomicrobiotas inhabiting animal skins/cuticles or plant phyllosphere have been unveiled with different features and biological functions, especially the defensive roles against different diseases [6–8]. For example, the protective roles of leaf microbiotas against bacterial or fungal pathogens have been evident in different plants such as *Arabidopsis thaliana*, rice, and citrus [9–11]. The microbes inhabiting insect cuticles can protect hosts from the topical infection of entomopathogenic fungi (EPF) like *Metarhizium anisopliae* and *Beauveria bassiana* [12–14]. Irrespective of their functional importance, plant or animal ectomicrobiome assembly is still elusive. The use of *Drosophila melanogaster* as a model for investigations has revealed that the ectomicrobiotas of adult flies were largely assembled from their fecal bacteria when insects were reared in vials to eat and excrete [15]. Metamorphic insects frequently molt, indicating that their cuticular microbiotas have to be quickly re-assembled after ecdysis. The beetle (*Lagria villosa*) larvae contain unusual pouch structures in cuticles that can house ectosymbionts for seeding bacterial cells after ecdysis [16]. The dynamic and function of insect cuticular microbiotas are still largely unclear, especially for those herbivorous insects.

The silkworm *Bombyx mori* is an important domesticated insect for the sericulture industry and a model animal for the research of caterpillar developments and life sciences [17, 18]. This oligophagous insect only feeds on mulberry (*Morus alba*) leaves and the change in diets, especially the use of artificial diet, could substantially change the gut microbiotas, growth, and physiology of silkworm larvae [19–23]. Silkworm mass rearing frequently encounters the threat of fungal, bacterial, virus, and microsporidian diseases [18]. Different bacteria isolated from silkworm gut have been found with probiotic potentials to improve insect resistance against diseases or toxic compounds including insecticides [24–27]. In contrast to the *per os* infection by pathogenic bacteria and viruses, fungal parasites infect insects via penetration of cuticles [28, 29]. Insect cuticular bacteria can thus mediate colonization resistance against EPF such as *M. robertsii* and *B. bassiana* by inhibiting fungal spore germination and or infection structure differentiation [6, 30]. The ectomicrobiotas of silkworm larvae are unclear and it is also unknown whether its cuticle bacteria could help improve insect survival against fungal parasite infections.

Co-existing microorganisms commonly engage in cross-inhibition and competition for resources by employing different strategies directed at each other. For example, to counteract the resistance of insect cuticular bacteria, *B. bassiana* secrets a defensin-like antibacterial peptide that can kill diverse bacteria, especially Gram-positive bacteria [31]. *M. robertsii*, however, produces different potent antibiotic compounds to combat different bacteria to facilitate fungal infection of insects or growth in diverse environments [30, 32]. Likewise, different bacteria can produce diverse antifungal metabolites or enzymes (e.g., lysozymes) [33]. Thus, species like *Bacillus* spp. have long been used as biocontrol agents against plant diseases, especially fungal pathogens [34, 35]. Similar to the uses for human health and the food industry, beneficial bacteria have also been explored as probiotics for promoting health in mass-reared insects [22, 36]. Until this study, it is unclear whether there are any probiotic bacteria that protect beneficial insects like silkworms against fungal pathogens entering from cuticles.

In this study, we investigated the age-associated ectomicrobiomes of silkworm larvae and found a quick increase in bacterial loads on larval cuticles after molting. One of the dominant bacteria *Mammaliicoccus sciuri* (*Ma. sciuri*, previously *Staphylococcus sciuri*) [37] isolated from silkworm cuticles could completely inhibit the spore germination of fungal pathogens *M. robertsii* and *B. bassiana* largely due to its secretion of an antifungal chitinolytic lysozyme. Interestingly, we found that *Ma. sciuri* originates from mulberry leaves that can be gathered by silkworms as an ecto- and endosymbiont to protect the caterpillars from fungal parasite infections.

## Materials and methods

### Fungal and bacterial strains

The wild-type strains of *M. robertsii* ARSEF 2575, *B. bassiana* ARSEF 2860, *Aspergillus oryzae* RIB 40, and *A. flavu*s NRRL 3357 were maintained on potato dextrose agar (PDA; BD Difco) at 25 ℃ for 2 weeks for harvesting conidial spores. Fungi were also grown in Sabouraud dextrose broth (SDB, BD Difco) for different experiments. The isolated *Ma. sciuri* strain CGMCC 28001 was grown on a Luria–Bertani (LB) plate at 25 ℃ or 30 ℃. The *Escherichia coli* strains BL21 and pLysS were maintained on LB plates and used for protein expression analysis.

### Silkworm rearing and surface bacterial isolation

The Nistari strain of *B. mori* larvae was conventionally reared (CR) with mulberry leaves at 25 ℃ and a relative humidity of 50%. Different instars/ages of larvae were then used for isolation of cuticular bacteria, including the end of the 4th instar larvae (L4E; stopping eating just before molting), and the newly molted (L5D0), three- (L5D3), and 6-day-old (L5D6) 5th instar larvae. To determine the resident or transient relationship of some bacteria, we also performed the diet switch experiments. Thus, silkworm larvae were fed with mulberry leaves to the end of the 4th instar, and the newly molted 5th instar insects were then fed with the bacterium-free Silkmate 2S fodder (Nosan Co., Yokohama, Japan) till the insect pupated. The collected insects were anesthetized on ice for 1 h, and three insects were used as a replicate by washing and vortexing in 50 ml phosphate buffer solution (PBS; pH, 7.0) for 30 s. The washing buffers were diluted properly (500 × for L4E and L5D6 samples; 100 × for L5D3 sample; no dilution for L5D0 sample) for inoculation of LB plates, Marine agar (Biofeng), and GYC plates (glucose, 50 g/l; yeast extract, 10 g/l; 0.5% CaCO_3_, 5 g/l; agar, 15 g/l) for 2 days at 30 ℃ to count bacterial colony forming units (CFUs), which were converted to the unit of CFUs per insect [31]. The antibiotic specifically inhibiting the Gram-positive (G + ; Nafcillin, Topscience, Shanghai) and Gram-negarive (G − ; Aztreonam, Qisong Biotech, Beijing) bacteria were added to LB medium at a final concentration of 50 μg/ml to determine the numbers of the G + /G − bacterial CFUs [30], respectively. There were 10 replicates for each age of insects, and a two-tailed Student’s *t-*test was conducted to compare the difference in CFU numbers between different ages of insects using the program GraphPad (ver. 10.1.1).

Individual colonies formed on LB plates were selected based on their phenotypes and transferred to new plates. Each bacterium was labeled and used for colony PCR using the primers 27F and 1492R1 (Table S1) [15]. PCR products were purified for Sanger sequencing and the obtained sequences were used for Blastn analysis to identify bacterial species.

### Microbiome analysis

The washing buffers obtained above were concentrated to 200 μl each by centrifugation at 12,000 rpm and 4 ℃ for 20 min. Each sample containing cuticular bacteria was treated with an equal volume of lysis buffer (Mouse Tissue Direct PCR Kit, Tiangen Biotech, Beijing) for 30 min at 65℃ and then 5 min at 95 ℃ [15]. The samples were then used as individual templates for PCR amplification of bacterial 16S rRNA genes with the universal primers 515F and 806R (Table S1) [25]. The purified products were then used to generate the amplicon libraries for sequencing (PE 2 × 250 bp; Illumina HiSeq 2500) at the service company Biozeron (Shanghai). Sequencing reads were normalized and clustered into individual operational taxonomic units (OTUs) at a cutoff of 97% identity [15]. The α-diversities of Shannon and Simpson indices were estimated based on the detected OTUs.

### Bacterial inhibition assays

The species of silkworm cuticular bacteria obtained above (Table S2) were used to test their inhibition effects on spore germination of *M. robertsii* and *B. bassiana* [31]. Each bacterial species was grown in the LB broth at 30 ℃ and 220 rpm overnight, and bacterial cells were concentrated by centrifugation. Bacterial cells (at a final OD600 = 0.01) and fungal spores (at the final concentration of 5 × 10^6^ conidia/ml) were mixed in 2 ml LB, and samples were transferred to individual Petri dishes (60 mm in diameter) and incubated at 30 ℃ for 16 h to determine the germination of *B. bassiana* and for 12 h for estimation of *M. robertsii* spore germinations. There were ten replicates for each bacterial species, and at least 100 fungal spores were counted for each replicate under microscopy.

To further test the inhibition effect of the selected *Ma. sciuri* strain, we conducted the growth inhibition assays on a solid LB medium. Thus, the sterile filter-paper strips (3 × 0.5 cm) were cut and transferred upside down to the right side of the LB plates (90 mm in diameter). The cells of *Ma. sciuri* (20 μl; OD 600 = 5) were inoculated on each strip, and fungal spores (10 μl; 5 × 10^6^ conidia/ml) were incubated on the left side of the same LB plates. The plates were incubated at 30 °C and photographed 8 and 16 days post-inoculations.

We analyzed and designed the specific primers V4F and V5R for targeting the 16S rDNA V4-V5 region of *Ma. sciuri* (Table S1). Primer specificity was verified by including the other isolated bacteria, such as *Glutamicibacter mishrai* (Actinomycetota, G +), *Enterobacter asburiae* (Gammaproteobacteria, G −), and *Agrobacterium larrymoorei* (Alphaproteobacteria, G −). After verification, primers were used to detect the presence or absence of *Ma. sciuri* on mulberry leaves and Silkmate fodder as well as the insect feces, guts, and cuticles of different age insects. Mulberry leaves were washed with sterile PBS buffer, and the washing buffers were concentrated by centrifugation and used for bacterial detection. The feces (20 each) were randomly collected into 1 ml PBS and suspended for 1 h before being used for bacterial detection.

### Antifungal component analysis

After the shaking growth of *Ma. sciuri* at 25 ℃ and 220 rpm for 12, 24, and 48 h, 1 ml bacterial culture was collected and centrifuged at 5000 rpm for 5 min. The supernatants were filtered through the sterile Millex® Syringe Filters (Merck) and diluted (1:20) into fresh LB broth to germinate fungal spores of *M. robertsii* and *B. bassiana*. Spore germinations in LB broth were included as mock controls. To determine the potential bioactive metabolite or protein/peptide factor in supernatants, we performed the batch growth of bacterial cells to a large volume (400 ml each) at 25 ℃ and 220 rpm for 24 h. After collecting the supernatant by centrifugation, an equal volume of ethyl acetate was used to extract small molecules by following a previous protocol [38]. LB broth without bacterial growth was extracted as a mock control. The concentrated extracts were dissolved in 2 ml methanol, which was added into LB broth at a ratio of 1:20 to inhibit the spore germination of both fungal parasites.

We also performed the precipitation of proteins/peptides from the aliquots of supernatants. Thus, the analytical ammonium sulfate was gradually added into the supernatant samples to a saturated level, and the precipitates were then collected by centrifugation. The samples were re-dissolved/suspended in 2.5 ml sterile PBS buffer and dialyzed in a dialysis tubing (1 kDa, Sigma-Aldrich) in 5 L PBS buffer three times by replacing the buffer every 8 h. The solutions were re-concentrated to 2.5 ml using a centrifugal evaporator (Martin Christ), and the samples were then loaded into individual Amicon® Ultra 10 K (Merck) devices to separate proteins (≥ 10 kDa) and peptides (≤ 10kDa) by centrifugation. The samples were lyophilized, and the proteins/peptides were added into LB broth at the final concentration of 10 and 100 μg/ml to examine their inhibition of fungal spore germination. The difference in inhibiting spore germinations was compared by two-tailed Student’s *t* test.

### Proteomic analysis

To identify the potential antifungal extracellular proteins, we conducted the proteomic analysis of the *Ma. sciuri* culture filtrates with and without co-culturing with fungi. The spores of *M. robertsii* and *B. bassiana* were inoculated in SDB and incubated at 25 ℃ and 220 rpm for 36 h. Mycelia were then collected by filtration, washed twice with sterile water, and used for co-culturing by sealing in dialysis tubes [39]. After growing *Ma. sciuri* in 400 ml LB broth at 25 ℃ and 220 rpm for 24 h, the dialysis tube containing 3.5 g of the fresh mycelia of *M. robertsii* or *B. bassiana* were added into the bacterial cultures for incubation for another 24 h. The *Ma. sciuri* cultures without fungi were incubated and included as controls. There were three replicates for each treatment. After incubations, fungal mycelial bags were carefully removed and the samples were centrifuged at 5000 rpm for 5 min. The supernatants were then used for protein precipitation with ammonium sulfate as described above. After extensive dialysis, the samples were lyophilized and used for proteomic analysis using the Q-Exactive mass spectrometer (Thermo Fisher Scientific) by the service company Omicsolution (Shanghai, China). A protein was considered identifiable with at least one unique peptide being detected in at least two replicates of each sample. Protein intensities were estimated by normalizing the spectral index for each protein [40]. The intensity data were processed by log 10 conversion and used for heat mapping analysis with GraphPad to show the differential expression of proteins among three treatments.

### Protein expression and antifungal activity assays

After proteomic analysis, three bacterial proteins showed upregulations in *Ma. sciuri* after being co-cultured with fungi and potential antifungal activities were expressed for activity assays, including WP_088592153 (termed Msp1), WP_088592189 (Msp2), and WP_088592490 (MsP3). The coding region of each protein gene was amplified using the 2 × Phanta Flash Master MixDye Plus kit (Vazyme) and different primer pairs (Table S1). After trial analysis, the purified products of *Msp1* and *Msp3* were cloned into the pET-28b plasmid by fusion with an MBP (maltose binding protein)-His (6 ×) tag at the C-termini, and *Msp2* was cloned into the pET-28b vector by fusion with a His (6 ×) tag at the C-terminus. The *Msp1* and *Msp3* expression vectors were transformed into the *E. coli* BL21 strain while the *Msp2* plasmid was transformed into the *E. coli* pLysS cells. *E. coli* cells were grown in LB and induced with 0.1 mM isopropyl β-D-1-thiogalactopyranoside (IPTG, Sigma-Aldrich) at 16 °C overnight. The fusion proteins were purified using the Ni–NTA beads (Smart-Lifescience). The MBP-His tag within the Msp1-MBP and Msp3-MBP fusion proteins was cleaved using a TEV protease. After cleavage, the product was purified again using the Ni–NTA beads, and the eluents were collected and dialyzed with a Tris buffer (pH = 8) to remove salts. The purified proteins were checked using a precast Omni-Easy™One-Step PAGE Gel Kit (15%, Epizyme) before use (10 and 100 µg/ml in LB) to examine their activities to inhibit *M. robertsii* spore germination.

### Chitinase activity assay

To determine the potential chitinase activity of lysozyme Msp1, we used the purified protein for activity assay using a Chitinase Assay Kit (Macklin). The chitinase from *Streptomyces griseus* (Sigma-Aldrich, C6137) was used as a positive control [41]. In addition, we tested whether Msp1 could hydrolyze colloidal chitin to form the hydrolytic zone in agar plates. Thus, chitin powder (5 g; Sigma-Aldrich) was first added to the concentrated HCl (37%; 60 ml) and stirred with a magnetic rod at 4 °C overnight. The samples were then washed with 95% ethanol twice followed by washing with sterile water before being embedded in water agars (5%, w/v). The plates were punched with holes (7.5 mm in diameter), and 20 μl PBS or Msp1 protein solution (100 μg/ml) were individually inoculated for 12 h.

### Cell damage assays

To determine whether Msp1 could damage fungal cells, we germinated the spores of *M. robertsii* (5 × 10^6^ conidia/ml) in LB with the addition of Msp1 at a final concentration of 10 µg/ml in a Petri dish (60 mm in diameter). The mock group was included without the addition of Msp1. Each group had three replicates. After 12 h, the supernatants were carefully removed with a pipette, and fungal cells were fixed in 4% formaldehyde for 12 h. The samples were then dehydrated for observation using the Field-Emission Scanning Electron Microscope (Merlin Compact VP, Zeiss) [42].

We also performed ninhydrin staining assays after treating *Metarhizium* spores with Msp1 [43]. The supernatants collected above were centrifuged at 12,000 rpm for 30 min, transferred, and added with PBS containing 2% (w/v) ninhydrin. A reference control group was included by only containing Msp1 protein (10 µg/ml). The samples were boiled in a water bath for 15 min, and immediately cooled on ice. Sample absorbance was measured at a wavelength of 570 nm with a spectrophotometer (GENESYS 50™, Thermo Fisher Scientific) [43].

### *Msp1* gene deletion and antifungal assays

We deleted the intact *Msp1* gene in *Ma. sciuri* using a CRISPR/Cpf1 technique for homologous recombination [44]. In brief, the flanking regions of *Msp1* were amplified using the primer pairs Msp1UF/Msp1UR and Msp1LF/Msp1LR (Table S1), respectively. The purified products were then fused into the *Xho1* restriction site of the pCpfSA vector using the Gibson Assembly Master Mix kit (NEB Biolabs) to generate the plasmid pCpfSA-Msp1. Five CRISPR RNAs (crRNAs) were synthesized by containing different protospacer adjacent motifs (PAMs) (Fig. S1; Table S1). The double-strand crRNAs were individually integrated into pCpfSA-Msp1 in a reaction system containing the *Bsal-*HF enzyme (NEB Biolabs) and T4 DNA ligase (Vazyme) [44]. The obtained plasmids were then used for individual transformation of the competent *Ma. sciuri* cells by electroporation at 1800–2100 kV (Bio-Rad MicroPulser #1652100).

The drug-resistant colonies were further transferred onto LB plates containing chloramphenicol (5 μg/ml) and verified by colony PCR. The deletion mutants were successfully obtained from crRNAs (Fig. S1) and used at different dosages (at final OD600 = 0.01, 0.05, 0.0025, and 0.00125, respectively) to examine together with the WT strain against the spore germination of *M. robertsii*.

### Protection of silkworm survival against fungal pathogen challenges

After preliminary trials, the newly-molted 5th instar CR silkworm larvae (10–12 h post-ecdysis) were pre-immersed in PBS buffer containing *Ma. sciuri* (OD 600 = 5) for 5 s. The control insects were immersed in PBS buffer without bacterial cells. After 24 h, insects were topically infected with the spore suspensions (5 × 10^7^ conidia/ml in 0.05% Tween 20) of either *M. robertsii* or *B. bassiana* by immersion for 30 s. Additional insects were immersed in 0.05% Tween 20 without fungal spores for 30 s as controls. Axenic silkworm larvae were also prepared by following a previous protocol [24]. In brief, the surface-sterilized silkworm eggs were hatched on and reared with the sterile diet of Silkmate 2S to the 5th instar. Food changings were conducted in a fume hood. Before using for gnotobiotic bioassays, individual larvae were homogenized with a sterile set of mortar and pestle. The samples were inoculated on LB plates to examine whether there was any bacterial growth. In addition, the homogenates were used as a template for PCR (2 × Phanta Flash Master Mix, Vazyme) examination of bacterial 16S rDNA using the conventional primers 518F and 1492R2 (Table S1). The confirmed axenic silkworm larvae were then used for gnotobiotic survival assays with the *M. robertsii* and *B. bassiana* spore suspensions similarly as described above. Both the CR and axenic silkworm larvae were also pretreated with the WT and Δ*Msp1* of *Ma. sciuri* (OD600 = 5) for 24 h before immersion with the spore suspensions of *M. robertsii*. The mortality was recorded every 12 h. There were 50 silkworm larvae used for each group, and those that died accidentally within 24 h after immersion were excluded in further analysis. The experiments were repeated at least three times, and the representative results from the same batch of experiments were shown. The log-rank test was conducted to compare the survival differences between treatments [45].

### Determination of *Ma. sciuri* on silkworm development and food protection

We soaked mulberry leaves in PBS containing the *Ma. sciuri* cells (OD600 value = 0, 0.01, 0.1, 1, and 5, respectively) for 5 s. After treatments, mulberry leaves were air dried to feed the newly molted 5th instar CR silkworm larvae till the insect was pupated. The sterile Silkmate diet was also mixed with 50 ml (per 100 g) of PBS containing the *Ma. sciuris* cells at the OD600 values of 0, 0.01, 0.1, 1, and 5, respectively. The fodders were then used to feed the axenic 5th instar larvae before they stopped eating for pupation. Each batch of the treated leaves or Silkmate was used within 2 days. Five days post-insect pupation, individual cocoons, and pupae were weighted to compare the differences between treatments by one-way ANOVA test using the Tukey method with GraphPad.

During the mass rearing of silkworms, artificial feeds can be frequently contained by *Aspergillus* fungi [20]. The species *A. oryzae* and *A. flavus* were thus used in the inhibition of spore germination with *Ma. sciuri* (OD600 = 0.01) for 12 h. In addition, the sterile Silkmate fodder (50 g) was added with an equal volume (10 ml each) of *Ma. sciuri* (OD600 = 5) and *Aspergillus* spore suspension (5 × 10^6^ conidia/ml). The samples without the addition of *Ma. sciuri* were included as controls. After transferring (10 g per plate) to the Petri dishes (120 mm in diameter), the samples were incubated at 30 ℃ for 10 days to determine the bacterial inhibition of aspergilli molding.

## Results

### Quick reassembling ectomicrobiotas after silkworm molting

**Fig. 1:**
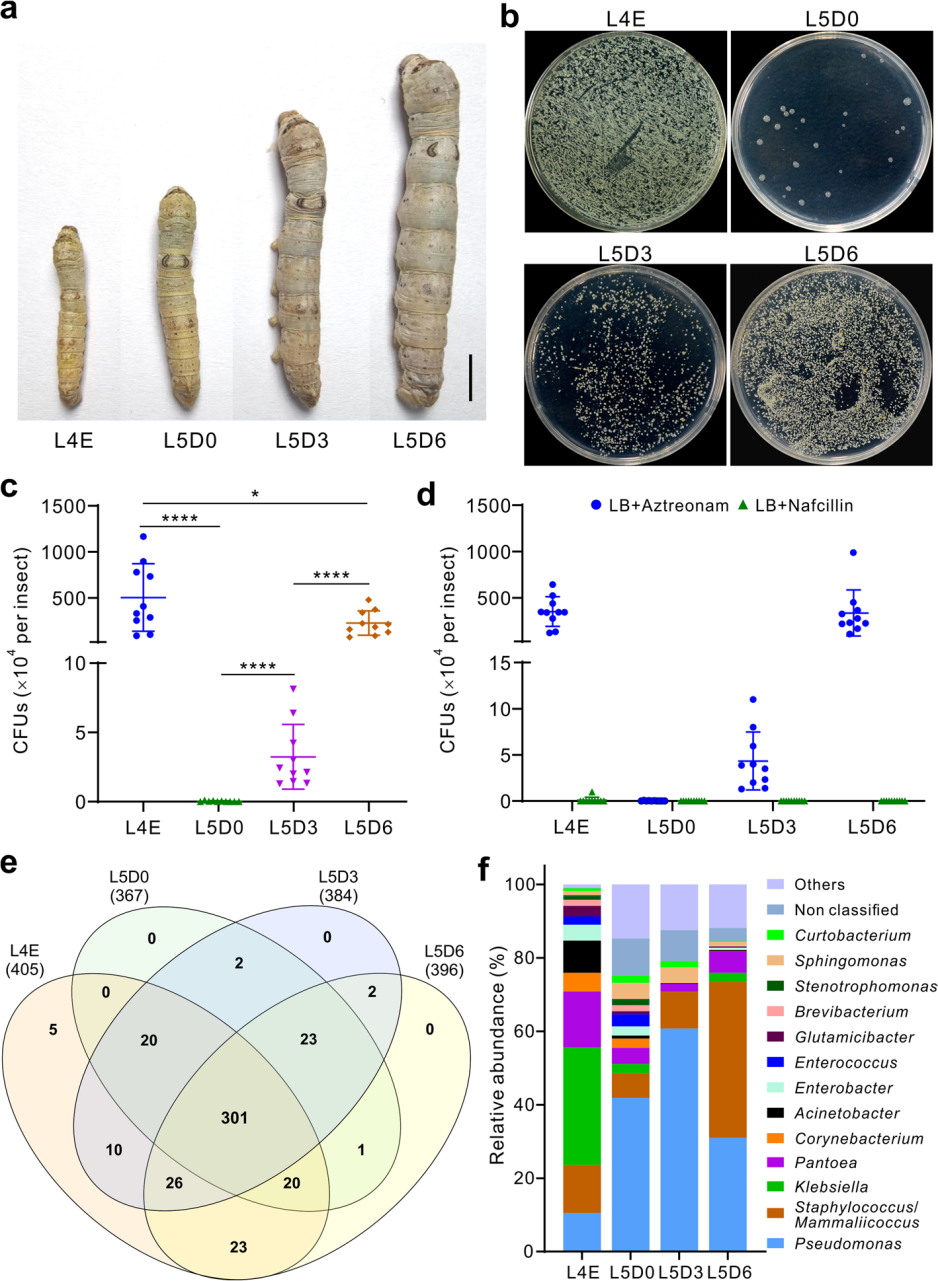
Quick re-assembly of ectomicrobiotas on silkworm cuticles after insect ecdysis. **a** Phenotype of the different ages of silkworm larvae used in experiments. The insects were conventionally reared with mulberry leaves. Bar, 1 cm. **b** Colony-forming patterns of the bacteria washed off from the different ages of silkworm larvae. **c** Comparison of the silkworm cuticular bacterial CFUs formed on LB agars among the different ages of silkworm larvae. Two-tailed Student’s *t-*test was conducted between samples: *, *P* < 0.05; ***, *P* < 0.001; ****, *P* < 0.0001. Ten independent replicates (three insects per replicate) were included for each sample. **d** The G + bacteria largely inhabiting silkworm larvae body surfaces. LB agars were added with the antibiotic nafcillin for suppressing the G + bacteria whereas the addition of aztreonam for inhibiting the G − bacteria. **e** Venn diagram analysis showing the overlap of bacterial OTUs among samples. **f** Variation of the OTU relative abundance at the bacterial genus level among silkworm ectomicrobiotas. L4E, the end of the 4th instar larvae; L5D0, the newly molted 5th instar larvae; L5D3 and L5D6, the third-day and sixth-day old of the 5th instar larvae

We examined the dynamics of silkworm cuticular microbiotas by washing the L4E, L5D0, L5D3, and L5D6 larvae that were conventionally reared (CR) with mulberry leaves (Fig. 1a). Plating washing buffer on LB agar and counting bacterial CFUs indicated that silkworm molting could substantially wipe off cuticular bacteria. However, bacterial loads could be quickly and increasingly re-assembled on cuticles after ecdysis (Fig. 1b, c). For example, the average bacterial numbers increased more than 70-fold in 3 days from 3.2 × 10^4^ CFUs per L5D3 insect to 2.3 × 10^6^ CFUs per L5D6 insect (*t*-test, *P* < 0.0001). The bacterial load differences could be similarly evident when plating the washing buffers on the marine agar (Fig. S2a) or GYC agar (Fig. S2b). After adding the G + (nafcillin) or G − (aztreonam) specific antibiotic in LB agar [15], bacterial growth and CFU counting revealed that the culturable G + bacteria largely inhabited silkworm cuticles (Fig. 1d).

The washing buffer samples were also used for the PCR amplification of the bacterial 16S rDNA, and the amplicons were used for the generation of sequencing libraries. The obtained microbiome data revealed that the bacterial OTUs were largely shared among different instars and ages of silkworms (Fig. 1e). Intriguingly, a rather similar number of OTUs was detected on the L5D0 silkworms and other ages of insects. However, the Shannon and Simpson diversity indices of the L5D0 insects were lower (*P* < 0.01) than those of the other age silkworms (Fig. S2c,d). It was unexpected to find that, except for a few specific OTUs for the L4E insects, the 5th instar larvae did not have any age-specific OTUs, indicating that bacterial number rather than bacterial species increase occurred on silkworm cuticles. The estimation of relative OTU abundance at the genus level indicated that the different ages of silkworm larvae had rather divergent abundance of bacteria (Fig. 1f). For example, different from the above colony plating assays, the G − *Klebsiella* bacteria dominated on the L4E insect surfaces whereas the G − *Pseudomonas* bacteria largely inhabited the 5th instar larvae. Otherwise, the G + *Staphylococcus/Mammaliicoccus* bacteria were rich in each stage of insects, especially in the L5D6 larvae.

### Identification of an antifungal bacterium from silkworm cuticles

While counting the CFUs mentioned above, individual colonies were transferred to new plates, and the 16S rDNAs of 29 randomly selected colonies were sequenced (Table S2). The non-redundant bacteria were then used in the inhibition of spore germination assays against both *M. robertsii* and *B. bassiana* by co-culturing with the bacterial cells in the LB broth [15]. It was found that *Ma. sciuri* could completely inhibit the spore germination of both fungi (Figs. S3a, b and S4a). A strain of *Serratia ureilytica* could completely suppress the germination of *B. bassiana* spores but not those of *M. robertsii* (Fig. S3a, b). We further performed the confrontation tests on LB agars between the selected bacteria and two fungi, which indicated again that *Ma. sciuri* but not the other bacteria could considerably suppress the growth of both *M. robertsii* and *B. bassiana* (Fig. S4b).

Previous studies have shown that *S. sciuri* (now *Ma. sciuri*) is widely present in soils, plant leaves, and humans as a commensal bacterium [46, 47]. This bacterium has also been detected in silkworm gut microbiomes [20, 21, 24]. We designed the specific primers for detecting *Ma. sciuri* (Fig. S5a), and found that this bacterium was present on mulberry leaves used for feeding silkworms. Unsurprisingly, *Ma. sciuri* was present on the cuticles and in the gut and feces of silkworm larvae fed with mulberry leaves (Fig. S5b). This bacterium was absent in the artificial Silkmate diet, and the fodder-fed silkworms were therefore not inhabited by *Ma. sciuri* (Fig. S5c). We also performed the diet-switching experiments after feeding insects with mulberry leaves to the end of the 4th instar, the newly molted 5th instar insects were then fed with Silkmate fodder. It was found that *Ma. sciuri* could still be detected from the feces, guts, and cuticles of the 5th instar silkworms before pupation (Fig. S5d). The data indicated therefore that this plant-derived bacterium could be a resident rather than a transient member on and in silkworms.

### Extracellular protein(s) of *Ma. sciuri* mediates the inhibition of fungal spore germination

**Fig. 2:**
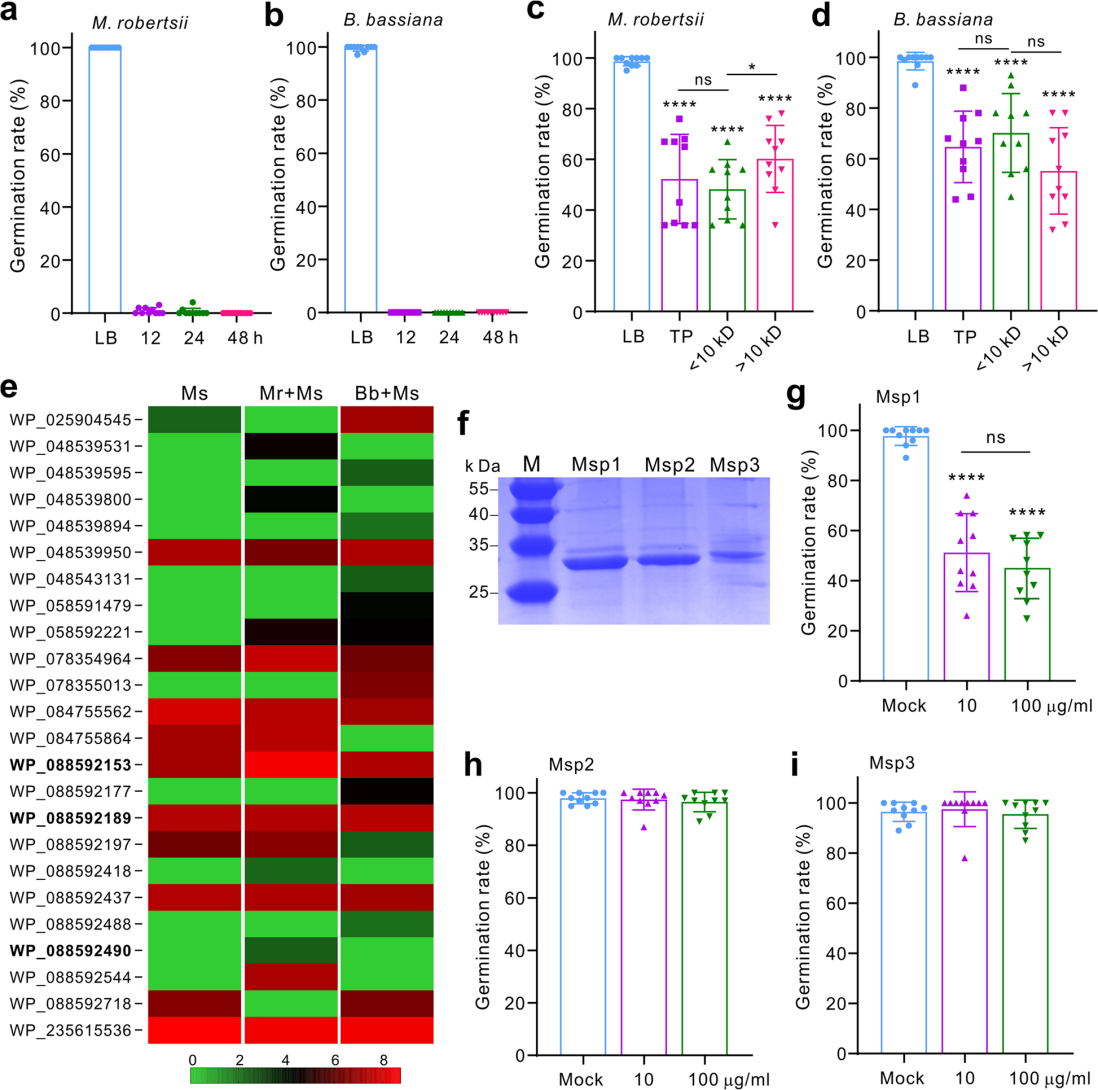
Inhibition of fungal spore germination by bacterial extracellular proteins. **a**, **b** Inhibition of *M. robertsii* (**a**) and *B. bassiana* (**b**) spore germination by the *Ma. sciuri* culture filtrates. *Ma. sciuri* was grown in the LB broth for the indicated times before being centrifuged, and the supernatants were used for germinating fungal spores. Germination of fungal spores in LB was included as mock controls. **c**, **d** The precipitated extracellular proteins of *Ma. sciuri* could inhibit the *M. robertsii* (**c**) and *B. bassiana* (**d**) spore germination. TP, total protein; Peptides (< 10 kD), and proteins (> 10 kD) were prepared using centrifugal filter units. **e** Heat mapping shows the differential expression of the *Ma. sciuri* extracellular proteins in different treatments. Ms, the proteins extracted from the pure *Ma. sciuri* culture. Mr + Ms, the proteins extracted from the *M. robertsii* and *Ma. sciuri* co-culture; Bb + Ms, the proteins extracted from the *B. bassiana* and *Ma. sciuri* co-culture. The protein intensity was obtained from three independent replicates by Log10 conversion. The proteins highlighted in bold were upregulated in challenging with fungi and selected for *E. coli* expression and antifungal activity assay. **f** Gel analysis of three expressed proteins. **g**–**i** The expressed Msp1 (**g**) but not Msp2 (**h**) and Msp3 (**i**) proteins could significantly inhibit the germination of *M. robertsii* spores. Panels **c**, **d**, **g** two-tailed Student’s *t-*test was conducted between each sample and mock control: ****, *P* < 0.0001. ns, not significant. Ten independent replicates were included for each sample

We next aimed to determine the antifungal factor(s) of *Ma. sciuri* by growing the bacterium in LB for 12, 24, and 48 h. The supernatants were then diluted (1:20) with fresh LB broth to test the spore germination of both fungi. It was found that the diluted culture supernatants could completely inhibit fungal spore germination (Fig. 2a, b), indicating that the potent antifungal component(s) was produced and secreted by *Ma. sciuri* into cultures. We then grew *Ma. sciuri* to a large volume for metabolite extraction and extracellular protein precipitation. The precipitated proteins were further separated into the aliquots of < 10 kD and > 10 kD using centrifugal filter units. The following experiments revealed that the ethyl acetate extracts could not inhibit the spore germination of both *M. robertsii* and *B. bassiana* (Fig. S3c, d). In contrast, the precipitated total proteins, small peptides (< 10 kD), or bigger proteins (> 10 kD) could similarly reduce the spore germination rate of *M. robertsii* (*P* < 0.0001) by about 50% and that of *B. bassiana* (*P* < 0.0001) by about 40% when compared with the mock control (Fig. 2c, d). The results indicated that the protein(s) instead of small molecules secreted by *Ma. sciuri* largely mediated the antifungal activity.

### A chitinolytic lysozyme produced by *Ma. sciuri* can damage fungal cells

To further determine the antifungal protein(s), we conducted the parallel growth of *Ma. sciuri* (Ms) without or with the mycelial cultures of *M. robertsii* (Ms + Mr) and *B. bassiana* (Ms + Bb), respectively. The total proteins of each sample were then precipitated, dialyzed, and subjected to mass spectrometry analysis. Different bacterial proteins (25 in total) were detected as well as three *Metarhizium* proteins from the Ms + Mr sample and ten *Beauveria* proteins from the Ms + Bb sample (Dataset S1). Differential expression of bacterial proteins was detected among three samples (Fig. 2e). Three proteins, i.e., WP_088592153 [Msp1; signal peptides (SP), 1–27 amino acids (aa)], WP_088592189 (Msp2; SP, 1–25 aa), and WP_088592490 (Msp3; SP, 1–24 aa), were selected for further activity assays based on the features of their expressions in *Ma. sciuri* pure culture, and upregulation by bacterium when being co-cultured with either fungus as well as the containing of putative antifungal/antibiotic domains. These three proteins each contain a SP region. Msp1 is similar (58% identity) to the lytic transglycosylase SceD of *S. aureus* [48], which contains a C-terminus lysozyme-like catalytic domain. Both Msp2 and Msp3 are the putative cysteine, histidine-dependent amidohydrolases/peptidase (CHAP) domain-containing proteins (Table S3). CHAP proteins also have lysozyme activities [49].

**Fig. 3:**
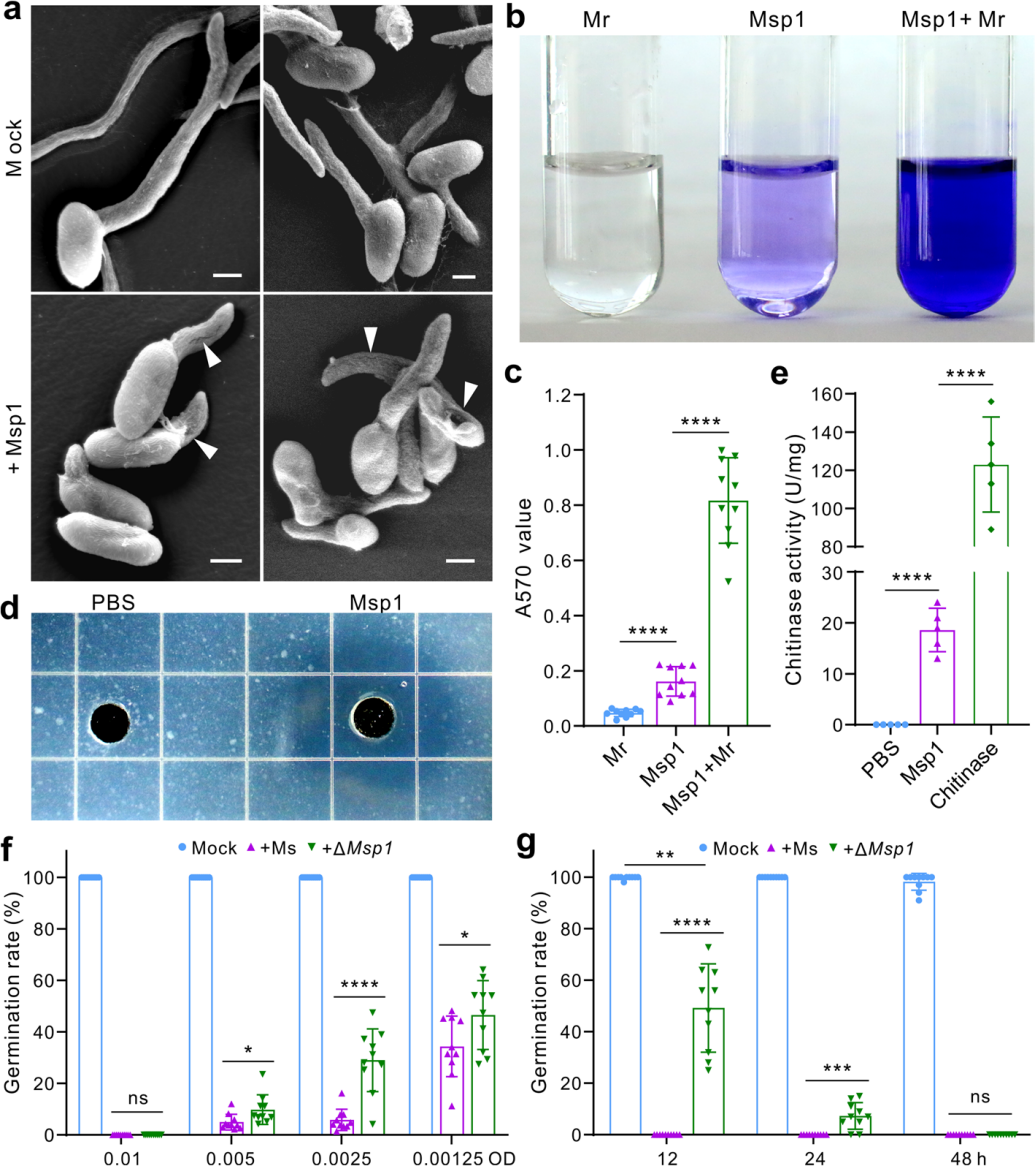
Disruption of fungal spores by Msp1 with a chitinase activity. **a** Disruption of *M. robertsii* spores by Msp1. The conidia of *M. robertsii* were incubated with or without Msp1 (at a final concentration of 10 μg/ml) in LB for 12 h before being fixed for SEM observation. Cells with lytic lesions are arrowed. Bar, 5 μm. **b** Examination of spore leakage after Msp1 treatment by ninhydrin staining. The conidia of *M. robertsii* (Mr, 5 × 10.^6^ conidia/ml) were suspended in sterile PBS buffer with or without Msp1 (10 μg/ml) for 12 h. The supernatant was then collected and treated with ninhydrin (2%, w/v). **c** Comparison of A570 absorbance after sample reaction with ninhydrin. **d** Formation of a chitin hydrolytic zone by Msp1. The water agar was prepared by containing 5% (w/v) colloidal chitin, and 20 μl of Msp1 (100 μg/ml) was loaded for 12 h. **e** Activity assays showing the chitinase activity of Msp1. The commercial chitinase isolated from *S. griseus* was used as a positive control. **f**, **g** Comparison of the inhibition of *M. robertsii* spore germination among the different dosage of WT *Ma. sciuri* (Ms) and Δ*Msp1* cells (**f**), and among LB culture filtrates (**g**). Panels **c**, **e**–**g** Two-tailed Student’s *t-*test was conducted between samples: ns, not significant; *, *P* < 0.05; ***, *P* < 0.001; ****, *P* < 0.0001

These three proteins were then heterologously expressed without their SPs in *E. coli*, and purified for antifungal activity assays (Fig. 2f). The use of two dosages (10 and 100 μg/ml) of each protein in LB for inhibition assays revealed that Msp1 but not Msp2 and Msp3 could significantly (*P* < 0.0001) inhibit the germination of the *M. robertsii* spores when compared with the mock control (Fig. 2g–i). Our further scanning electron microscopy (SEM) analysis revealed that Msp1 treatment could damage the cells of *M. robertsii* (Fig. 3a). Thus, the leakage of intracellular protein contents was detected by ninhydrin staining assays (Fig. 3b, c).

Considering that the lysozyme domain of lytic transglycosylase shows a chitinase activity [50], we tested the chitinolytic activity of Msp1. It was found that the addition of Msp1 into the colloidal chitin agar could result in the formation of a hydrolytic zone (Fig. 3d). Activity assay confirmed that Msp1 has a chitinase activity, however, which was lower (*P* < 0.0001) than that of a commercial chitinase purified from *Streptomyces griseus* (Fig. 3e).

### Deletion of *Msp1* reduced the antifungal ability of *Ma. sciuri*

To further verify the Msp1 antifungal activity, we performed the deletion of *Msp1* gene in *Ma. sciuri* using a CRISPR/Cpf1 technique [44], and the independent mutants were verified (Fig. S1). The subsequent uses of the wild-type (WT) and mutant isolates of *Ma. sciuri* for the inhibition of the *M. robertsii* spore germination revealed that Δ*Msp1* and WT could similarly inhibit spore germination when being co-cultured with fungal spores at a dosage of 0.01 OD600. However, the null mutant showed impaired antifungal inhibition ability at lower dosages compared to the WT treatments (Fig. 3f). We also used the culture broths of the WT and Δ*Msp1* of *Ma. sciuri* for assaying the inhibition of fungal spore germination. As a result, in contrast to the WT samples, the Δ*Msp1* culture broths of different fermentation times could not completely inhibit the germination of the *M. robertsii* spores until being cultured up to 48 h (Fig. 3g). Taken together, the data confirmed that Msp1 of *Ma. sciuri* plays an essential role in attacking fungal spores, and additional antifungal factor(s) could be produced by *Ma. sciuri* to synergistically suppress fungal spore germination.

### Improving silkworm survival against fungal infections by *Ma. sciuri*

**Fig. 4:**
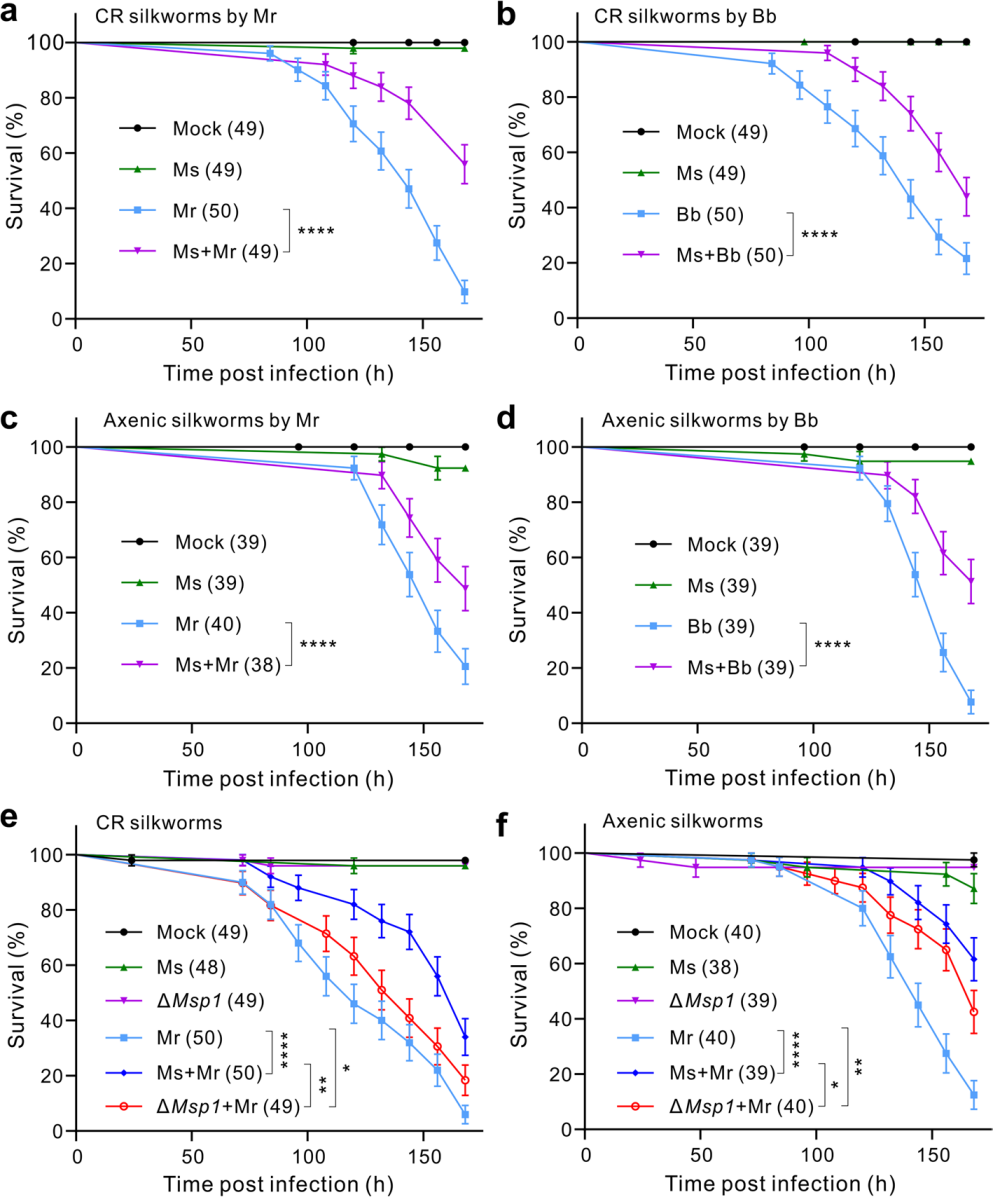
Protection of silkworm larvae by *Ma. sciuri* against fungal infections. **a**, **b** Protection of the conventionally reared (CR) silkworm larvae against *M. robertsii* (Mr, **a**) and *B. bassiana* (Bb, **b**) infection by *Ma. sciuri*. **c**, **d** Protection of the axenic silkworm larvae against *M. robertsii* (**c**) and *B. bassiana* (**d**) infection by *Ma. sciuri*. **e**, **f** Deletion of *Msp1* in *Ma. sciuri* impaired the bacterial protection of CR (**e**) and axenic (**f**) silkworm larvae against *M. robertsii* infection. The newly molted 5th instar larvae were immersed in *Ma. sciuri* (Ms) cells (OD600 = 5) for 5 s, and after 24 h the insects were topically treated with fungal spore suspensions (5 × 10^7^ conidia/ml in 0.05% Tween 20). Log-rank tests were conducted between the survival curves: *, *P* < 0.05; **, *P* < 0.01; ***, *P* < 0.001; ****, *P* < 0.0001. Mock control insects were treated with 0.05% Tween 20. The insects only treated with *Ma. sciuri* (Ms) were also included as controls. The number of insects finally included in analysis is shown in parentheses

We next performed silkworm survival assays with or without the pretreatment of insects using the *Ma. sciuri* cells for 24 h before the topical infections. It was found that the pretreatment of the CR larvae with bacterial cells could significantly increase insect survival against *M. robertsii* (*χ*^2^ = 23.7, *P* < 0.0001) and against *B. bassiana* (*χ*^2^ = 36.8, *P* < 0.0001) (Fig. 4a, b). We also generated the axenic silkworm larvae for gnotobiotic survival assays (Fig. S5e–g). It was found that the pretreatment of the germ-free silkworms with *Ma. sciuri* could also substantially (*P* < 0.0001) improve silkworm survival against either *M. robertsii* or *B. bassiana* infection when compared with those without the pretreatment of bacterial cells (Fig. 4c,d).

After the pretreatment of the CR and axenic silkworms with the WT and Δ*Msp1* of *Ma. sciuri*, comparative survival assays revealed that the deletion of *Msp1* could considerably attenuate the bacterial protection ability examined with both CR (*P* < 0.01) and axenic silkworms (*P* < 0.05) against *M. robertsii* (Fig. 4e,f). However, relative to the insects without pretreatments, the use of the Δ*Msp1* cells could still protect the CR (*P* < 0.05) and axenic (*P* < 0.01) silkworms against fungal infections. It is noteworthy that the sole treatment of silkworms with the *Ma. sciuri* cells had no negative effect on the survival of both the CR and axenic silkworms (Fig. 4).

### Probiotic effect of *Ma. sciuri* on protecting silkworm fodder

To further determine the probiotic effect of *Ma. sciuri*, we used the different dosages of bacterial cells to pretreat mulberry leaves and Silkmate fodder before use for feeding silkworm larvae. It was found that the pupae and cocoon weights of the CR and axenic insects had no obvious difference between the mock control and those reared with the diets pretreated with different amounts of bacterial cells (Fig. S6).

**Fig. 5:**
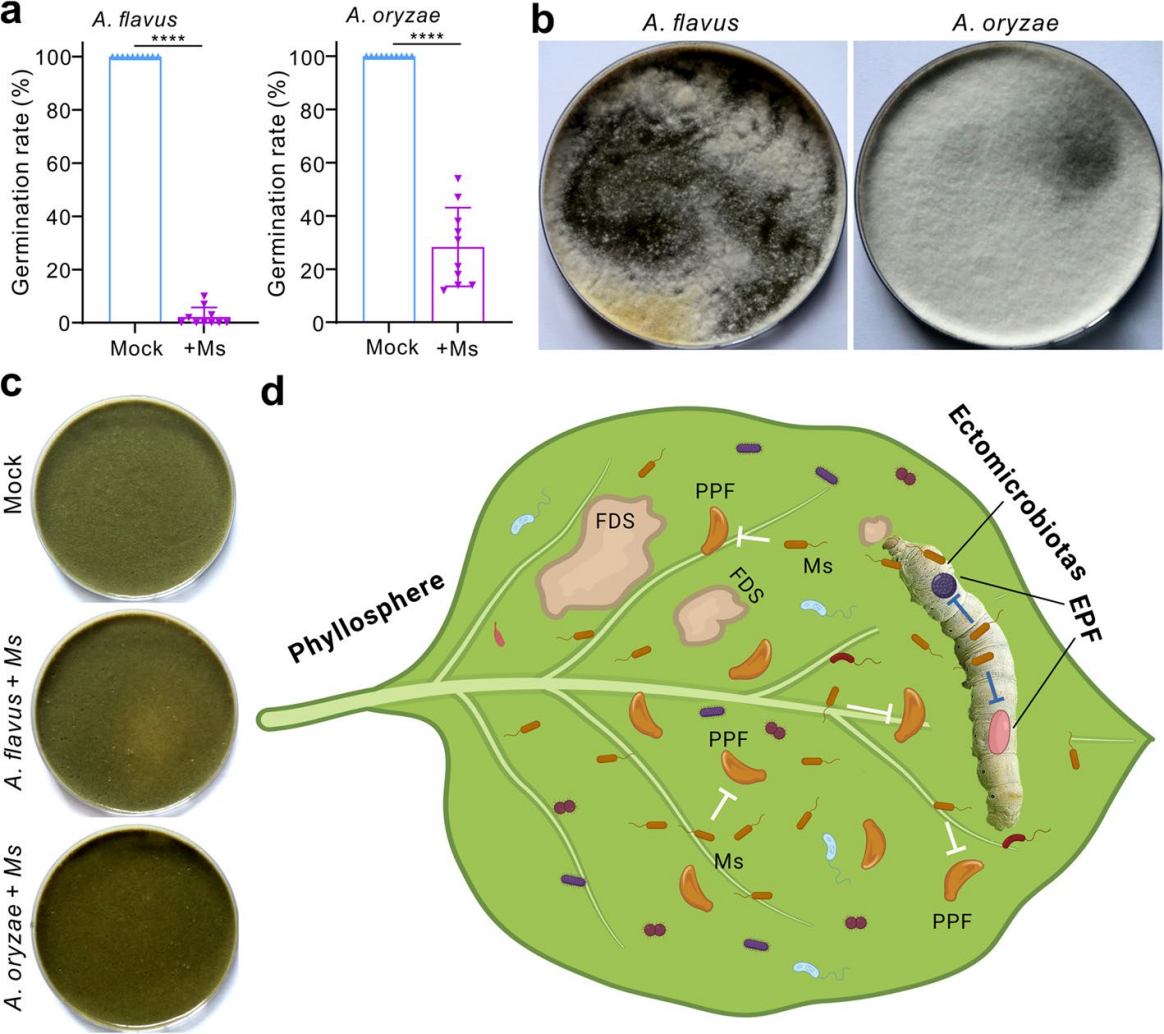
Probiotic effect of *Ma. sciuri* on diet protection and schematic transfer of phyllosphere bacteria to insects. **a** Differential inhibition of *Aspergillus* spore germination by *Ma. sciuri* (Ms). The bacterial cells were added at a dosage of OD600 = 0.01 for 12 h. **b** Molding of the sterile Silkmate fodders after inoculation of *A. flavus* and *A. oryzae* for 10 days. **c** Pretreatment of the sterile Silkmate fodders with the *Ma. sciuri* cells inhibits the growth of two *Aspergillus* fungi. The sterilized fodder was included as a mock control. The photos were taken 10 days post-inoculations. **d** Schematic transfer of phyllosphere bacteria to herbivorous insect cuticles that can protect insects against fungal parasites. EPF, entomopathogenic fungi; PPF, plant pathogenic fungi; FDS, fungal disease spot; Ms, *Ma. sciuri*

Having found that the artificial diets could be frequently contaminated by aspergilli molds, we tested and found that *Ma. sciuri* could also completely inhibit the germination of the *A. flavus* spores and substantially suppress (*P* < 0.0001) the germination of *A. oryzae* spores (Fig. 5a). We then tested the inoculation of the Silkmate fodder with *Aspergillus* spores and found that both *Aspergillus* species could outgrow on the fodders (Fig. 5b). However, the co-inoculation of fodder with aspergilli spores and *Ma. sciuri* cells could prevent diet molding by both fungi (Fig. 5c). The data indicate that the use of *Ma. sciuri* can additionally benefit the protection of silkworm diets against moldy fungi.

## Discussion

In this study, we found that the conventionally-reared (CR) silkworm larvae could quickly reassemble their ectomicrobiotas after molting including the gathering of mulberry leaf bacteria. Microbiome analysis revealed that the cuticles of the newly molted and different ages of insects largely shared the OTUs of different bacteria, and the *Pseudomonas*, *Mammaliicoccus*, and *Staphylococcus* bacteria quickly expanded on cuticles when silkworms fed on mulberry leaves. Taken together with the gnotobiotic survival assays, it was interesting to find that the silkworm cuticular bacterium *Ma. sciuri* that originated from mulberry leaves could confer defense against fungal parasite infections by secreting a chitinolytic antifungal lysozyme. The cross-kingdom transfer of the plant phyllosphere bacteria to herbivore cuticles can thus benefit insects in battling the infection of fungal pathogens (Fig. 5d).

It was unsurprising to find that the bacterial loads sharply dropped on the newly formed cuticles after silkworm molting. However, intriguingly, a roughly equal number of bacteria OTUs could be detected in the L5D0 insects. In contrast to the beetle larvae [16], the pouch-like structure is absent on silkworm larvae for housing bacteria during insect molting. It is possible but remains to be determined that the bacteria hiding on the trachea of silkworm larvae may contribute to the seeding of cuticular bacteria after insect molting [51]. Similar to the ingestion of plant bacteria into insect guts [5], the body surfaces of silkworm larvae can be “contaminated” with the mulberry leaf phyllosphere bacteria during insect feeding and crawling. Additional bacteria other than *Ma. sciuri* could be transferred to silkworm cuticles. It is known that the endo- or ecto-microbiotas of animals, especially the latter, could be contaminated with environmental bacteria [52]. In addition to the previous detection of *S. sciuri* (now *Ma. sciuri*) in silkworm gut microbiomes [20, 24], we found that *Ma. sciuri* were present on mulberry leaves, silkworm cuticles, guts, and feces. In contrast, *Ma. sciuri* was absent on and in silkworms after being fed with the bacterium-free fodder. The diet switched from mulberry leaves to an artificial diet without *Ma. sciuri* for the 5th instar larvae did not lead to the disappearance of this bacterium from silkworm cuticles and guts. Taken together, our data confirmed that silkworms gathered the antifungal *Ma. sciuri* from mulberry leaves as a symbiont to combat fungal pathogen infection (Fig. 5d). Silkworm has been domesticated for more than 5000 years [53], it is still elusive when silkworms evolved the ability to deploy this bacterium for disease resistance.

Phyllosphere microbiomes play an essential role in protecting plant health by antagonizing pathogenic bacteria and fungi [8, 54]. Apart from *Ma. sciuri*, we found that the isolated cuticular bacterium *Serratia ureilytica* could completely inhibit the germination of *B. bassiana* spores but not those of *M. robertsii*. Even the underlying mechanism remains to be determined, it is implicative that there would be additional cuticular bacteria other than *Ma. sciuri* involved in protecting silkworms against fungal pathogens. In support of this, different G + and G − bacteria isolated from *Drosophila* adult surfaces have been tested with varied but significantly inhibitory effects on the germination of *M. robertsii* and *B. bassiana* spores [15]. Likewise, the dominant G − *Pseudomonas* and *Serratia* bacteria isolated from the Japanese pine sawyer beetle (*Monochamus alternatus*) cuticles could completely inhibit the germination of *B. bassiana* spores [12]. The reciprocal evidence has shown that the deletions of the defensin-like antimicrobial peptide gene in *B. bassiana* and the antibiotic biosynthetic genes in *M. robertsii* impaired fungal ability to counterattack insect cuticular bacteria and therefore fungal virulence against insects during topical infections [30–32]. The protective effect of insect ectomicrobiotas against fungal pathogens would therefore be common [6], which might have facilitated the arms-race co-evolution among insect hosts, insect ectomicrobiotas, and fungal pathogens. It remains to be investigated in other insects in terms of the employment of the defensive microbes from phyllospheres by insect herbivores, especially those devastating agriculture pests, against EPF since which may impede the biocontrol efficacy of mycoinsecticides.

Antagonisms commonly occur between microbes while the strategies may vary from either side. To counterattack fungal competition, bacteria can produce antifungal compounds or peptides/enzymes [55, 56]. We found that a chitinolytic lysozyme Msp1 secreted by *Ma. sciuri* demonstrated an antifungal activity by damaging fungal cell walls. Since chitin is one of the critical cell wall components of fungi [57], it is thus not surprising to find that *Ma. sciuri* or its culture broth could similarly inhibit the spore germination of aspergilli fungi. Different lysozymes with chitinolytic activity have also been identified from other bacteria such as *Ralstonia* and *Streptomyces* spp., which have the potential to produce N-acetyl chitooligosaccharides from chitin materials [58, 59]. Otherwise, the antibacterial and or antifungal lysozymes can be produced by different plants and animals for immune defenses against different pathogens, some of which have been used as antibiotic agents in the food industry [60]. We examined and found that the chitinase activity of Msp1 was significantly lower than that of the commercial *Streptomyces* chitinase, which could explain, at least in part, why the deletion of *Msp1* did not completely disable the antifungal activity of *Ma. sciuri*. It was found that a serine protease produced by the biocontrol bacterium *Bacillus amyloliquefaciens* had a broad spectrum of antifungal activity [61]. Serine proteases were also detected in the culture broth of *Ma. sciuri*. It remains to be determined whether these proteases and or other enzyme(s)/factors are used by *Ma. sciuri* to maintain the synergistic antifungal activity. For example, interestingly, it was found that the volatile compounds produced by *S. sciuri* had an antifungal activity and potential against the strawberry anthracnose fungi [62].

Consistent with the antifungal activity of *Ma. sciuri*, our fungal infection assays confirmed that the mono-association of bacterial cells with either the CR or axenic silkworm larvae could significantly improve silkworm survival against the infection of *M. robertsii* or *B. bassiana*. Similarly, a lactic bacterium (*Lactobacillus paraplantarum*) was found with a probiotic potential after feeding to promote silkworm immunity against the pathogenic bacterium *Pseudomonas aeruginosa* [63]. We also found that the pretreatment of mulberry leaves or artificial fodder with *Ma. sciuri* had no negative effect on insect development, which was consistent with a previous finding that a gut-isolated strain of *S. sciuri* did not affect silkworm growth [24]. Taken together with the probiotic effect of *Ma. sciuri* on diet protection, our results indicate that the use of this bacterium may benefit the industrial and mass rearing of silkworms using artificial diets.

In conclusion, we report that the deployment of mulberry leaf bacteria to cuticles by silkworms can benefit the insects to combat fungal parasite infections. We also unveil that the antifungal bacterium *Ma. sciuri* can use an extracellular chitinolytic lysozyme to damage fungal cells. In addition to revealing the cross-kingdom transfer of beneficial bacteria from plants to insects, the beneficial bacterium identified in this study has the potential to be used for safer sericulture.

### Supplementary Information

**Additional file 1: Figure S1.** Screening of *Msp1* deletion in *Ma. sciuri *by colony PCR (a) and verification by sequencing of PCR products (b). The putative mutant 3 (mut3) was not a successful gene deletion mutant. PAM, protospacer adjacent motif within the used CRISPR RNAs (crRNAs). **Figure S2.** Silkworm cuticular bacterial CFU counting and estimation of bacterial OTU diversity. a, b Comparison of the cuticular bacterial CFUs formed on the marine agar (a) and GYC medium (b) among the different ages of silkworm larvae. Two-tailed Student’s *t-*test was conducted between samples: *, *P *< 0.05; ***, *P* < 0.001; ****, *P *< 0.0001. c, d Comparison of the Shannon (c) and Simpson (d) diversity indices among the ectomicrobiotas of the different ages of silkworm larvae. One-way ANOVA analysis was conducted to compare difference between samples: the column labelled with different capital letters, *P *< 0.01; different lower letters, *P* < 0.05. Ten independent replicates (three insects per replicate) were included for each sample.** Figure S3.** Screening and evaluation of bacteria isolated from silkworm surfaces for antifungal activity. a, b Inhibition or non-inhibition of *M. robertsii* (a, for 12 h) and *B. bassiana* (b, for 16 h) spore germination by different bacteria isolated from silkworm cuticles. Fungal spores (5 × 10^6^ conidia/ml) were germinated in LB with the addition of bacterial cells each at 0.01 OD600. c, d The ethyl acetate extracts of *Ma. sciuri* have no effect on inhibiting *M. robertsii* (c) and *B. bassiana *(d) spore germination. *Ma. sciuri* was inoculated in LB for 24 h and the cultures were centrifuged, and both the supernatant and bacterial cells were extracted and used for inhibition assays. **Figure S4.** Inhibition of fungal spore germination and growth by *Ma. sciuri*. a Microscopic images showing the inhibition or non-inhibition of *Metarhizium* and *Beauveria *spore germination by different bacteria. Fungal spores (5 × 10^6^ conidia/ml) were co-incubated with bacterial cells (each at a final value of OD600 = 0.01) in LB broth for 12 h (*M. robertsii*) or 16 h (*B. bassiana*) prior to imaging. Spore germination in LB was used as a mock control. Ungerminated fungal spores are arrowed. Bar, 5 μm. b Confrontation test of bacterial inhibition or non-inhibition of fungal growth for different times. Fungi were inoculated with 1 μl of spore suspensions (1 × 10^6^ conidia/ml each). The bacterial strips were prepared by soaking the filter-paper strips in bacterial cells (OD600 = 5) for 30 sec before inoculation. The strips without bacterial cells were used as mock controls. DPI, day post inoculation. **Figure S5.** PCR verification of the presence of different bacteria. a Verification of the specific PCR primers for detecting *Ma. sciuri *(*Ma. sc*). Different isolates of *Ma. sciuri* are as shown in Table S1. The bacterial species *Glutamicibacter mishrai* (*G. mi*), *Enterobacter asburiae *(*E. as*), and *Agrobacterium larrymoorei* (*A. la*) were included as negative controls. b Verification of the presence of *Ma. sciuri* on mulberry leaves and leaf-fed silkworms. c Verification of the absence of *Ma. sciuri* in Silkmate fodder and fodder-fed silkworms. L4S, slough of the 4^th^ instar larvae. d PCR verification of the symbiotic presence of *Ma. sciuri* in the 5^th^ instar silkworms fed with the bacterium-free fodder. The insects were fed with mulberry leaves till the end of the 4^th^ instar. e Images showing the silkworms conventionally reared (CR) with mulberry leaf and axenically reared with sterile Silkmate fodder. f, g Verification of the obtained axenic silkworm larvae by PCR of bacterial 16S rDNA (f) and plating of insect homogenates for bacterial colony formation (g). The CR silkworms were used as a positive control. **Figure S6.** Pretreatment of silkworm diets using the *Ma. sciuri* cells has no negative effect on insect development. a, b Feeding of the 5^th^ instar larvae with the mulberry leaves soaked in *Ma. sciuri* cells (from 0.01 – 5 OD600 in sterile BPS buffer) has no obvious negative effect on the pupa (a) and cocoon (b) weight of silkworms. c, d Feeding of the 5^th^ instar larvae with the artificial Silkmate fodder added with different amount of *Ma. sciuri* cells has no obvious negative effect on the pupa (c) and cocoon (d) weight of silkworms. One-way ANOVA analysis was conducted: ns, not significant. **Table S1.** Primers used in this study. **Table S2.** Isolation of cuticular bacteria from silkworm larvae. **Table S3.** Mass spectrometry detection of culture filtrate proteins secreted by *Ma. sciuri*.

**Additional file 2: Dataset S1.** LC-MS proteomic analysis of the supernatant proteins isolated from *Ma. sciuri *(Ms) after co-culturing without or with *M. robertsii *(Mr) or *B. bassiana* (Bb).

## Data Availability

The SRA data of silkworm microbiome analysis have been deposited in the NCBI database with an accession number PRJNA911351 (SRR22731736-SRR22731691).

## Abbreviations

EPF: Entomopathogenic fungi
CR: Conventionally reared
CFU: Colony forming unit
OTU: Operational taxonomic unit
G +: Gram-positive
G −: Gram-negative
SP: Signal peptide
WT: Wild type
