# Supplementary material for: From phyllosphere to insect cuticles: silkworms gather antifungal bacteria from mulberry leaves to battle fungal parasite attacks
Source: Microbiome. 2024 Feb 26;12:40. doi: 10.1186/s40168-024-01764-6 (PMC10895815; doi:10.1186/s40168-024-01764-6)
Supplement: Supplementary file 2 — Additional file 1: Figure S1. Screening of Msp1 deletion in Ma. sciuri by colony PCR (a) and verification by sequencing of PCR products (b). The putative mutant 3 (mut3) was not a successful gene deletion mutant. PAM, protospacer adjacent motif within the used CRISPR RNAs (crRNAs). Figure S2. Silkworm cuticular bacterial CFU counting and estimation of bacterial OTU diversity. a, b Comparison of the cuticular bacterial CFUs formed on the marine agar (a) and GYC medium (b) among the different ages of silkworm larvae. Two-tailed Student’s t-test was conducted between samples: *, P < 0.05; ***, P < 0.001; ****, P < 0.0001. c, d Comparison of the Shannon (c) and Simpson (d) diversity indices among the ectomicrobiotas of the different ages of silkworm larvae. One-way ANOVA analysis was conducted to compare difference between samples: the column labelled with different capital letters, P < 0.01; different lower letters, P < 0.05. Ten independent replicates (three insects per replicate) were included for each sample. Figure S3. Screening and evaluation of bacteria isolated from silkworm surfaces for antifungal activity. a, b Inhibition or non-inhibition of M. robertsii (a, for 12 h) and B. bassiana (b, for 16 h) spore germination by different bacteria isolated from silkworm cuticles. Fungal spores (5 × 10^6^ conidia/ml) were germinated in LB with the addition of bacterial cells each at 0.01 OD600. c, d The ethyl acetate extracts of Ma. sciuri have no effect on inhibiting M. robertsii (c) and B. bassiana (d) spore germination. Ma. sciuri was inoculated in LB for 24 h and the cultures were centrifuged, and both the supernatant and bacterial cells were extracted and used for inhibition assays. Figure S4. Inhibition of fungal spore germination and growth by Ma. sciuri. a Microscopic images showing the inhibition or non-inhibition of Metarhizium and Beauveria spore germination by different bacteria. Fungal spores (5 × 10^6^ conidia/ml) were co-incubated with bacterial cells (each at [file 40168_2024_1764_MOESM1_ESM.pdf]

Supporting Figures

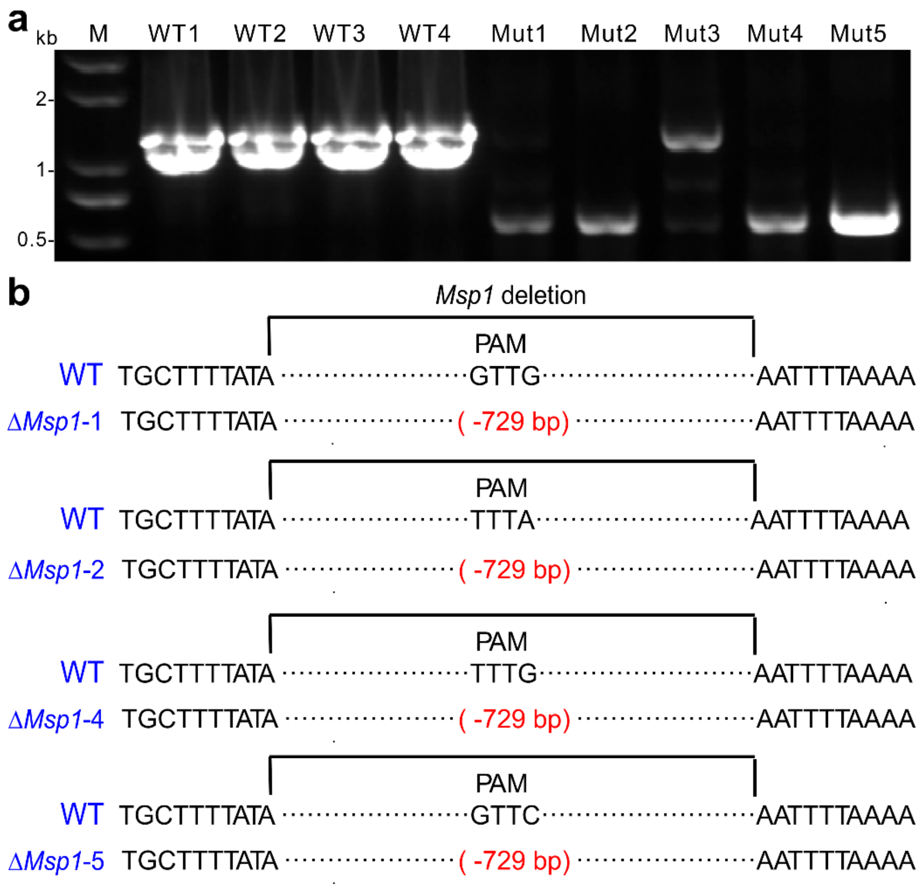

Fig. S1 Screening of *Msp1* deletion in *Ma. sciuri* by colony PCR (**a**) and verification by sequencing of PCR products (**b**). The putative mutant 3 (mut3) was not a successful gene deletion mutant. PAM, protospacer adjacent motif within the used CRISPR RNAs (crRNAs).

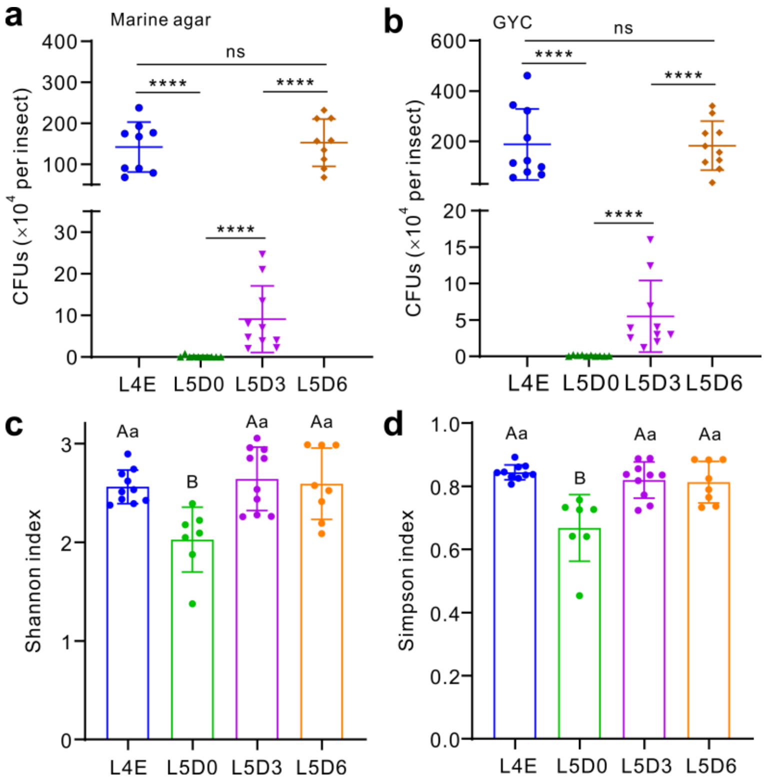

Fig. S2 Silkworm cuticular bacterial CFU counting and estimation of bacterial OTU diversity. **a, b** Comparison of the cuticular bacterial CFUs formed on the marine agar (**a**) and GYC medium (**b**) among the different ages of silkworm larvae. Two-tailed Student's *t*-test was conducted between samples: *, $P < 0.05$; ***, $P < 0.001$; ****, $P < 0.0001$. **c, d** Comparison of the Shannon (**c**) and Simpson (**d**) diversity indices among the ectomicrobiotas of the different ages of silkworm larvae. One-way ANOVA analysis was conducted to compare difference between samples: the column labelled with different capital letters, $P < 0.01$; different lower letters, $P < 0.05$. Ten independent replicates (three insects per replicate) were included for each sample.

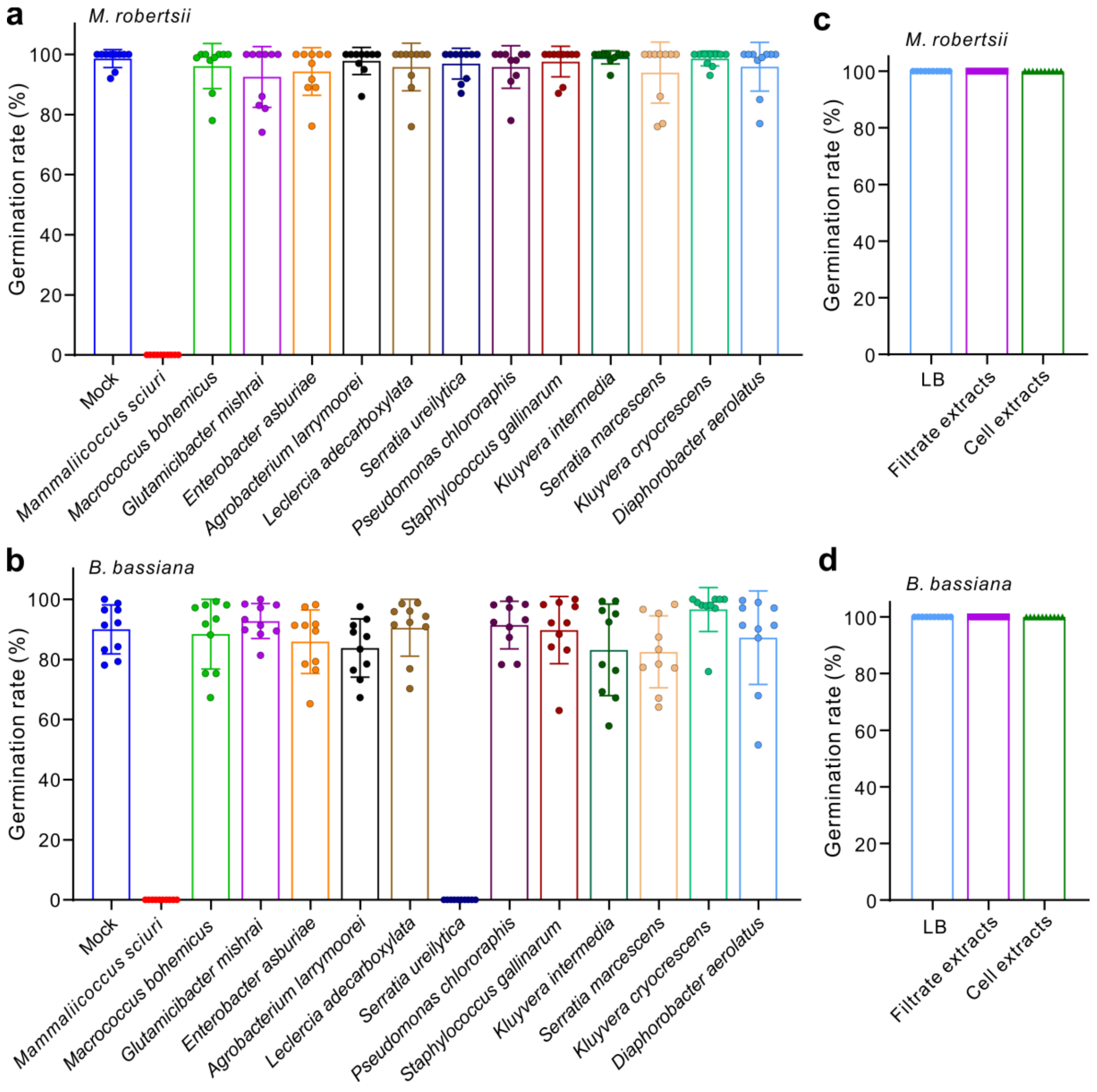

Fig. S3 Screening and evaluation of bacteria isolated from silkworm surfaces for antifungal activity. **a, b** Inhibition or non-inhibition of *M. robertsii* (**a**, for 12 h) and *B. bassiana* (**b**, for 16 h) spore germination by different bacteria isolated from silkworm cuticles. Fungal spores (5×10^6 conidia/ml) were germinated in LB with the addition of bacterial cells each at 0.01 OD600. **c, d** The ethyl acetate extracts of *Ma. sciuri* have no effect on inhibiting *M. robertsii* (**c**) and *B. bassiana* (**d**) spore germination. *Ma. sciuri* was inoculated in LB for 24 h and the cultures were centrifuged, and both the supernatant and bacterial cells were extracted and used for inhibition assays.

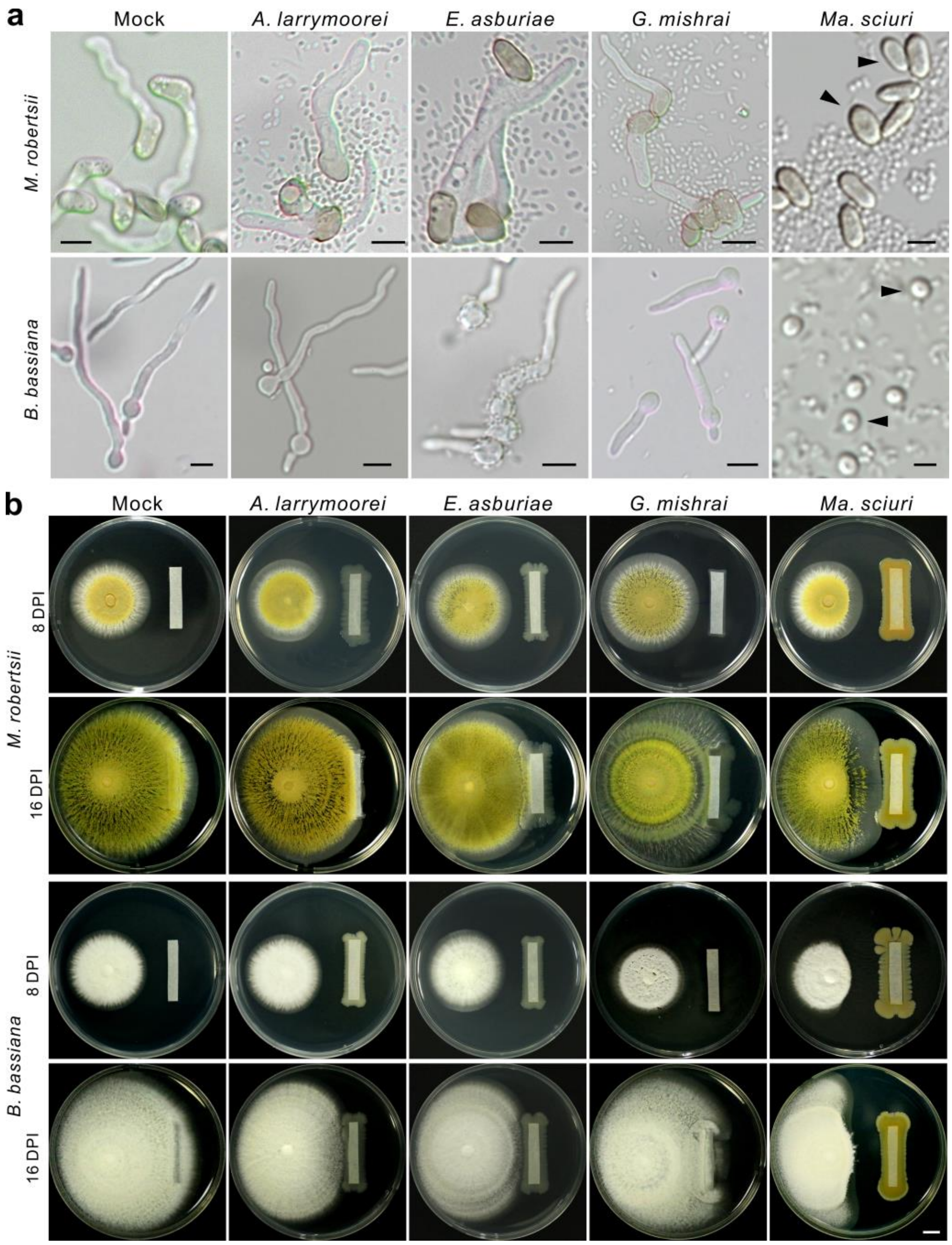

Fig. S4 Inhibition of fungal spore germination and growth by *Ma. sciuri*. **a** Microscopic images showing the inhibition or non-inhibition of *Metarhizium* and *Beauveria* spore germination by different bacteria. Fungal spores (5×10^6 conidia/ml) were co-incubated with bacterial cells (each at a final value of OD600 = 0.01) in LB broth for 12 h (*M. robertsii*) or 16 h (*B. bassiana*) prior to imaging. Spore germination in LB was used as a mock control. Ungerminated fungal spores are arrowed. Bar, 5 μm. **b** Confrontation test of bacterial inhibition or non-inhibition of fungal growth for different times. Fungi were inoculated with 1 μl of spore suspensions (1×10^6 conidia/ml each). The bacterial strips were prepared by soaking the filter-paper strips in bacterial cells (OD600 = 5) for 30 sec before inoculation. The strips without bacterial cells were used as mock controls. DPI, day post inoculation.

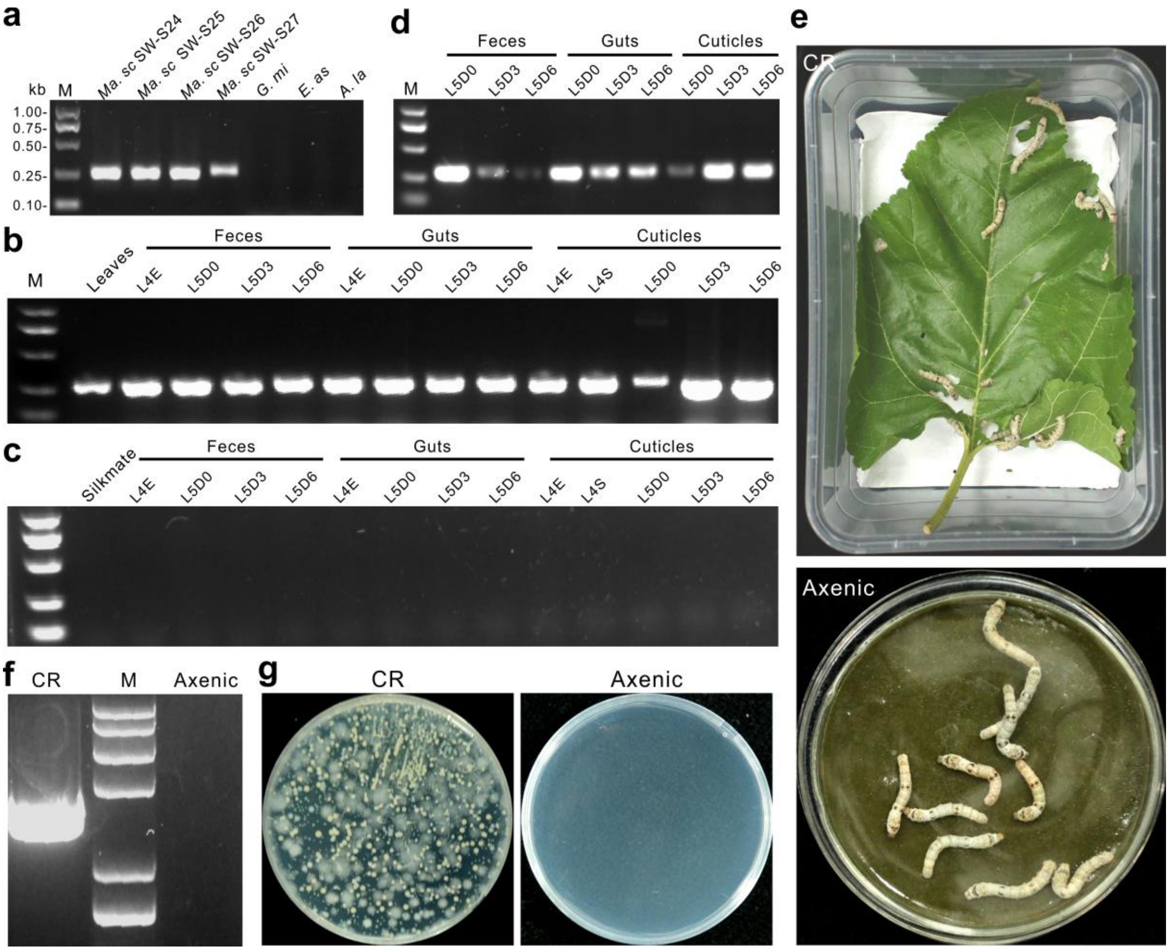

Fig. S5 PCR verification of the presence of different bacteria. **a** Verification of the specific PCR primers for detecting *Ma. sciuri* (*Ma. sc*). Different isolates of *Ma. sciuri* are as shown in Table S1. The bacterial species *Glutamicibacter mishrai* (*G. mi*), *Enterobacter asburiae* (*E. as*), and *Agrobacterium larrymoorei* (*A. la*) were included as negative controls. **b** Verification of the presence of *Ma. sciuri* on mulberry leaves and leaf-fed silkworms. **c** Verification of the absence of *Ma. sciuri* in Silkmate fodder and fodder-fed silkworms. L4S, slough of the 4th instar larvae. **d** PCR verification of the symbiotic presence of *Ma. sciuri* in the 5th instar silkworms fed with the bacterium-free fodder. The insects were fed with mulberry leaves till the end of the 4th instar. **e** Images showing the silkworms conventionally reared (CR) with mulberry leaf and axenically reared with sterile Silkmate fodder. **f, g** Verification of the obtained axenic silkworm larvae by PCR of bacterial 16S rDNA (**f**) and plating of insect homogenates for bacterial colony formation (**g**). The CR silkworms were used as a positive control.

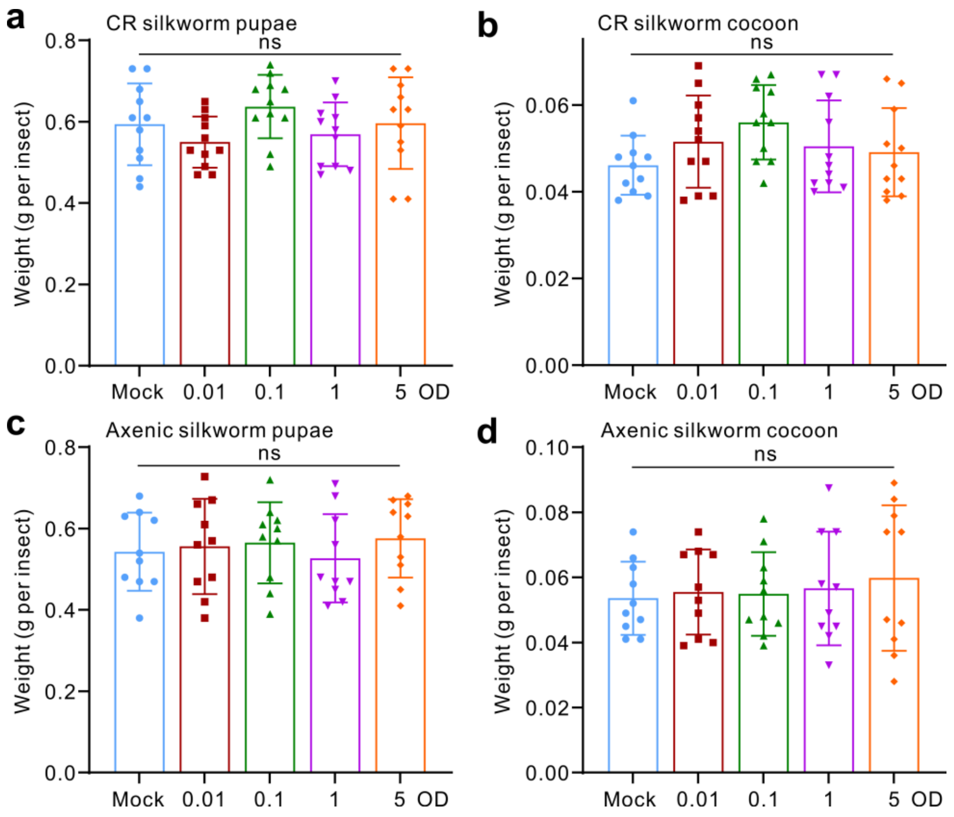

Fig. S6 Pretreatment of silkworm diets using the *Ma. sciuri* cells has no negative effect on insect development. **a, b** Feeding of the 5th instar larvae with the mulberry leaves soaked in *Ma. sciuri* cells (from 0.01 – 5 OD600 in sterile BPS buffer) has no obvious negative effect on the pupa (**a**) and cocoon (**b**) weight of silkworms. **c, d** Feeding of the 5th instar larvae with the artificial Silkmate fodder added with different amount of *Ma. sciuri* cells has no obvious negative effect on the pupa (**c**) and cocoon (**d**) weight of silkworms. One-way ANOVA analysis was conducted: ns, not significant.

Supporting Tables

Table S1. Primers used in this study.

Primers	Primer sequence (5′-3′)*	Note
806R	GGACTACNNGGGTATCTAAT	16S rDNA amplification
515F	GTGCCAGCMGCCGCGG	
1492R1	TACGGYTACCTTGTTACGACTT	
27F	AGAGTTTGATCMTGGCTCAG	
518F	GTATTACCGCGGCTGCTGG	
1492R2	CGGTTACCTTGTTACGACTT	
V4F	TGATGTGAAAGCCCACGGCTCAAC	Specific for detecting *Ma. sciuri*
V5R	CACTAAGGGGCGGAAACCC	
Msp1R	TATTTCCAAGGTTCT GCTGAACAGGACGTAAATAAAGAGCA	Msp1 expression
Msp1F	GGCCGCAGAGTCGAC TTATGCAGTTACCCATTGTGAAGCG	
Msp2R	AGAAGGAGATATACCATGGGCTCAACTCAACATACTGT AAAAAATGG	Msp2 expression
Msp2F	GTGGTGGTGCTCGAG ATGAATATATGAATAGTTATATACTTGTGA	
Msp3R	TATTTCCAAGGTTCT GCTGATATTAAAGTAAAAGAAGGACAAT	Msp3 expression
Msp3F	GGCCGCAGAGTCGAC CTAATGAATATAATTATATGAACCAGCTGC	
Msp1UF	GCTAACCACGCCGCTTTTTTACGTCTGCATCTAGAATG CAAGGAGTTGGTTGATATGAATATTGGAGCT	*Msp1* deletion
Msp1UR	ATTCGGAGGGTTTTAAAATTTATAAAAGCAGTATTAAAA TTTTATTCAACACATTAATCCAACGCC	
Msp1LF	ATTTTAATACTGCTTTTATAAATTTTAAAACCCTCCGAAT AATTTTTTATTTATAGTATCTTTTTTAAGA	
Msp1LR	ACCTTACCAGAGGGCGCCCCAGCTGGCAATTCCGACGA TCGTAAGCTTTAAGAATTTGTGCTTTTGAAATGT	
CrRNA1	TGAAGCGCCTGCAGT**GTTG**TATA	CrRNA used for *Msp1* deletion
CrRNA2	ATTGGTA**TTTA**CCTGCTGCACCT	
CrRNA3	TCTGATTGGAA**GTTA**TACTCGTA	
CrRNA4	TG**TTTG**CTGCTGGAGCTGGAGTT	
CrRNA5	ATTAA**GTTC**TGGTGCATTAGATT	

*, the nucleotides bolded in CrRNAs showing the PAM motifs.

Table S2. Isolation of cuticular bacteria from silkworm larvae.

**Clone No.**	**Species**	**16S rDNA top hit**
SW-S1	*Agrobacterium larrymoorei*	NR_026519.1
SW-S2	*Aureimonas* sp.	KY653043.1
SW-S3	*Aureimonas* sp.	KY653043.1
SW-S4	*Brachybacterium* sp.	JQ359088.1
SW-S5	*Diaphorobacter aerolatus*	NR_133739.1
SW-S6	*Enterobacter asburiae*	NR_024640.1
SW-S7	*Enterobacter* sp.	LC484760.1
SW-S8	*Enterococcus mundtii*	MW135232.1
SW-S9	*Enterococcus mundtii*	MW135243.1
SW-S10	*Glutamicibacter arilaitensis*	MG788347.1
SW-S11	*Glutamicibacter mishrai*	NR_169398.1
SW-S12	*Kluyvera cryocrescens*	NR_114043.1
SW-S13	*Kluyvera intermedia*	NR_041327.1
SW-S14	*Leclercia adecarboxylata*	NR_104933.1
SW-S15	*Macrococcus bohemicus*	NR_159093.1
SW-S16	*Methylobacterium komagatae*	MT386294.1
SW-S17	*Microbacterium proteolyticum*	MT993604.1
SW-S18	*Pseudomonas chlororaphis* sub sp. *aurantiaca*	NR_043935.1
SW-S19	*Pseudomonas poae*	MT631989.1
SW-S20	*Rhodococcus erythropolis*	KF358249.1
SW-S21	*Serratia marcescens*	NR_028802.1
SW-S22	*Serratia ureilytica*	NR_042356.1
SW-S23	*Staphylococcus gallinarum*	NR_036903.1
**SW-S24***	***Mammaliicoccus sciuri***	**NR_025520.1**
SW-S25	*Mammaliicoccus sciuri*	NR_025520.1
SW-S26	*Mammaliicoccus sciuri*	NR_025520.1
SW-S27	*Mammaliicoccus sciuri*	KP400531.1
SW-S28	*Staphylococcus succinus*	MN826566.1
SW-S29	*Staphylococcus xylosus*	MH210865.1

*, this strain has been deposited at China General Microbiological Culture Collection Center (CGMCC 28001) and used in this study.

Table S3. Mass spectrometry detection of culture filtrate proteins secreted by *Ma. sciuri*.

Accession	Average log10 (peak intensity)			Description
	**Ms**	**Ms + Mr**	**Ms + Bb**	
WP_025904545	2.25	0.00	7.09	type I glyceraldehyde-3-phosphate dehydrogenase
WP_048539531	0.00	4.54	0.00	hypothetical protein
WP_048539595	0.00	0.00	2.37	alanine dehydrogenase
WP_048539800	0.00	4.24	0.00	cytochrome b5
WP_048539894	0.00	0.00	1.94	hypothetical protein
WP_048539950	7.29	6.35	7.32	polyglycerol-phosphate lipoteichoic acid synthase
WP_048543131	0.00	0.00	2.36	aconitate hydratase
WP_058591479	0.00	0.00	4.24	L-glutamate gamma-semialdehyde dehydrogenase
WP_058592221	0.00	4.78	4.41	5'-nucleotidase lipoprotein e(P4) family
WP_078354964	6.62	7.74	6.27	CHAP domain-containing protein
WP_078355013	0.00	0.00	6.52	glycine C-acetyltransferase
WP_084755562	8.00	7.48	7.11	serine protease
WP_084755864	7.18	7.54	0.00	LysM domain-containing peptidoglycan hydrolase
**WP_088592153**	**7.16**	**8.56**	**7.36**	**Lytic transglycosylase family lysozyme**
WP_088592177	0.00	0.00	4.48	L-glutamate gamma-semialdehyde dehydrogenase
**WP_088592189**	**7.44**	**7.39**	**7.51**	**CHAP domain-containing protein**
WP_088592197	6.26	6.71	2.41	CHAP domain-containing protein
WP_088592418	0.00	2.18	0.00	cytochrome c oxidase subunit II
WP_088592437	7.44	7.36	7.08	glucosaminidase domain-containing protein
WP_088592488	0.00	0.00	2.05	phosphoglycerate kinase
**WP_088592490**	**0.00**	**2.35**	**0.00**	**CHAP domain-containing protein**
WP_088592544	0.00	7.27	0.00	hypothetical protein
WP_088592718	6.63	0.00	6.42	trypsin-like serine protease
WP_235615536	8.74	8.51	8.50	M4 family metallopeptidase
